# Discrimination of dissociated lymphoma cells from leukocytes by Raman spectroscopy

**DOI:** 10.1038/s41598-020-72762-5

**Published:** 2020-09-25

**Authors:** Yuko Iwasaki, Masahiko Kawagishi, Hiroshi Takase, Kyoko Ohno-Matsui

**Affiliations:** 1 Department of Ophthalmology and Visual Science, Tokyo Medical and Dental University, 1-5-45 Yushima, Bunkyo-ku, Tokyo, 113-8519 Japan; 2 Department of Neuroanatomy and Cellular Neurobiology, Tokyo Medical and Dental University, 1-5-45 Yushima, Bunkyo-ku, Tokyo, 113-8519 Japan

**Keywords:** Cancer screening, Diagnostic markers, Eye diseases

## Abstract

Diagnosis of intraocular lymphoma is difficult. Among the hurdles in the diagnosis are the variety of reactive inflammatory and ischemic changes among intraocular lymphoma patients. Thus, a novel diagnostic method is desired such that lymphoma cells can be distinguished by the signals intrinsic to the cells, not by those from the surrounding tissues with reactive changes. Raman spectroscopy is a technique that can detect intrinsic signals from each cell. Therefore, Raman spectroscopy is a good candidate for an intraocular evaluation technology that could contribute to improve the diagnosis of intraocular lymphoma. In this study, we tested whether the intrinsic Raman signals from malignant lymphoma cells, in the absence of surrounding tissue, were sufficient for the discrimination of malignant lymphoma cells from leukocytes. We acquired spectra from dissociated lymphoma cells, along with spectra from normal B cells and other leukocytes involved in intraocular inflammatory diseases. We analysed the spectra using principal component analyses and quadratic discriminant analyses. We found that Raman spectra from dissociated cells without confounding tissues showed high discriminating ability, regardless of the variation due to day-to-day differences and donor differences. The present study demonstrates the possible effectiveness of Raman spectroscopy as a tool for intraocular evaluation.

## Introduction

Raman spectroscopy detects the vibrations of chemical bonds in molecules; this technology has been widely used from basic to clinical research fields. A Raman spectrum of biomedical samples exhibits a combination of multiple Raman peaks that represent metabolic and chemical conditions of tissues or cells. Because the technique does not require sample labelling, Raman spectroscopy is a promising method for in vivo evaluation of human tissues in clinical and medical settings. Many papers have reported the application of in vivo Raman spectroscopy to skin cancer diagnosis, and the technique’s usefulness also has been evaluated in various clinical settings^1–3^. In addition, the technology is reported to be useful for detecting brain, nasopharyngeal, oral, and lung cancers^4–7^. Desroches et al*.* reported that targeted brain cancer tissue biopsy is possible using in vivo Raman spectroscopy^8^. Other applications, such as in vivo monitoring of human cervix throughout pregnancy and of glucose concentration in blood, also have been reported^9, 10^. In ophthalmology, only resonant Raman spectroscopy has been used for in vivo imaging, because the laser intensity needs to meet the safety criteria for eye evaluation^11^. Recently, Stiebing et al. have shown that non-resonant spontaneous Raman spectroscopy, employing a laser with an intensity weak enough to meet the safety criteria, could detect a Raman spectrum from the retina^12^. Therefore, it is expected that in vivo Raman spectroscopy also might be of use in the field of ophthalmology.

**Figure 1:**
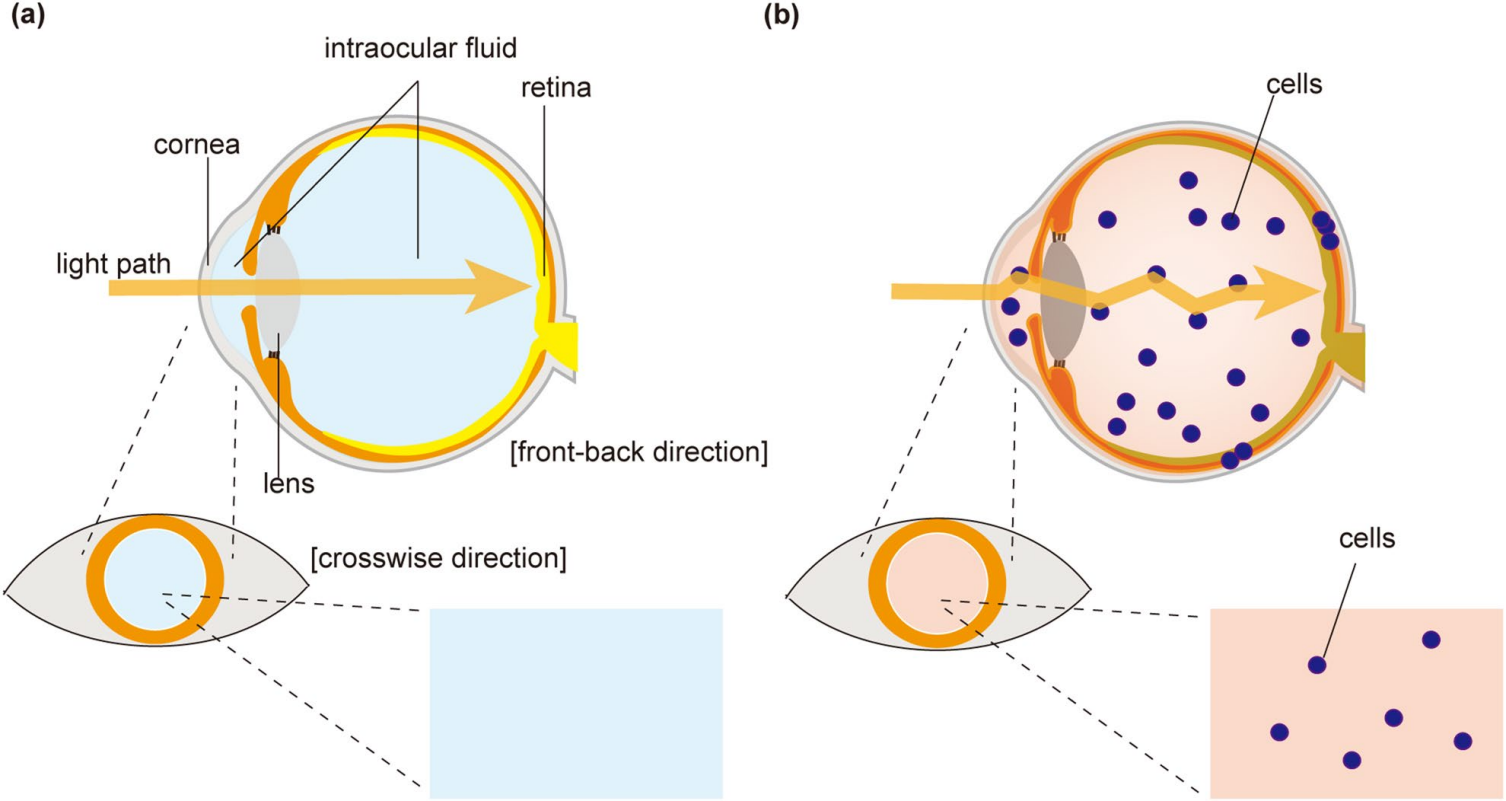
The transparent structure of the eye facilitates the use of optical methods for the examination of cells in the eye. (**a**) Schematic of the eye. Light reaches the retina through transparent eye structures such as the cornea, lens, and intraocular fluid. The retina converts light into electrical impulses. Under normal physiological conditions, very few cells are observed in the intraocular fluid. (**b**) When cells invade the eye, it is possible to observe cells optically. Cell types differ among various conditions such as infectious, inflammatory, and malignant diseases.

Among the major organs in human, the eye is a particularly good candidate for optical observation because the anterior parts of the eye structure are transparent, consistent with the need to pass light to the retina, where the stimulus is converted into electric neural impulses (Fig. 1a). In previous studies, Raman spectroscopy has been used for evaluation of gross eye structures such as retina, lens, and cornea^11–13^. However, the utility of Raman spectroscopy for characterizing infiltrating cells that invade the intraocular fluid remains unknown (Fig. 1b).

These infiltrating cells are observed in the cases of intraocular infectious, inflammatory, or malignant diseases. Such infiltration impedes the transparency of eye structures (Fig. 1b), resulting in decreased vision in patients. Notably, the type of the infiltrating cells varies among different diseases. In intraocular lymphoma, a diagnosis is made by cytological confirmation of the presence of malignant lymphoma cells. These lymphoma cells also invade the central nervous system at a high rate (56–90%); the prognosis of patients with intraocular lymphoma therefore is poor, with median survival times of 58 months^14–16^. Although an early diagnosis is desirable, the actual diagnostic process often is time-consuming. In fact, it previously has been reported that the delay between onset of symptoms and diagnosis is 4–40 months for intraocular lymphoma^15^. Cytological evaluation has been employed as a standard diagnostic method, but this technique presents several practical difficulties. First, decision-making regarding the surgical collection of cytological samples is difficult and can take months. Second, the accurate diagnosis rate by conventional cytology is low (30–40%)^17, 18^, possibly due to the small volume and the low cellularity of the eye sample as well as the fragility of the tumour cells. Therefore, multiple cytology-based technologies have been employed to improve the diagnostic rate, including the cell block technique, intraocular fluid cytokine analysis, and PCR for detection of immunoglobulin gene rearrangement^15^. Despite these efforts, a universal procedure with a high diagnostic rate has not been established to date.

Alternatively, technologies that can evaluate cells in the patient eye without requiring surgery can be exploited to overcome the difficulties in cytology mentioned above. Indeed, various technologies have been applied for intraocular evaluation and continue to play an important role in understanding miscellaneous eye conditions. For example, laser flare photometry and retinal angiography detect vessel barrier leakage resulting from inflammatory cytokine elevation in the eye. Angiography also can detect abnormal angiogenesis due to severe ischemia. However, such conventional methods are not as useful for lymphoma diagnosis. One possible reason is that these technologies primarily detect secondary environmental changes due to the malignant cell invasion, such as inflammatory reactions and ischemic changes, rather than the characteristics of the invading cells. Such intraocular environmental changes are diverse even among patients with the same disease, which limits the diagnostic application of these technologies. Therefore, we sought to apply a novel technology that can evaluate the characteristics of the invading cells themselves for the diagnosis of intraocular lymphoma.

As a candidate technique for intraocular evaluation, we focused on Raman spectroscopy for its ability to optically detect the chemical and metabolic conditions of cell samples. Raman spectroscopy previously has been reported to be useful for the diagnosis of malignant diseases, including malignant lymphoma^19–21^. However, those earlier reports evaluated lymph node and spleen sections that included surrounding cells and stroma, meaning that the acquired data could have been confounded by characteristics of the deteriorating surrounding tissues. Therefore, it was not clear whether Raman signals would permit discrimination of malignant lymphoma from other diseases based on properties intrinsic to the lymphoma cells. To address this question, we tested whether the Raman scattering properties of malignant cells without the surrounding tissues were sufficient for the discrimination of malignant lymphoma. Specifically, we examined the potential effectiveness of Raman spectroscopy when applied for intraocular evaluation.

## Results

### Discrimination of lymphoma cells from B lymphocytes

**Figure 2:**
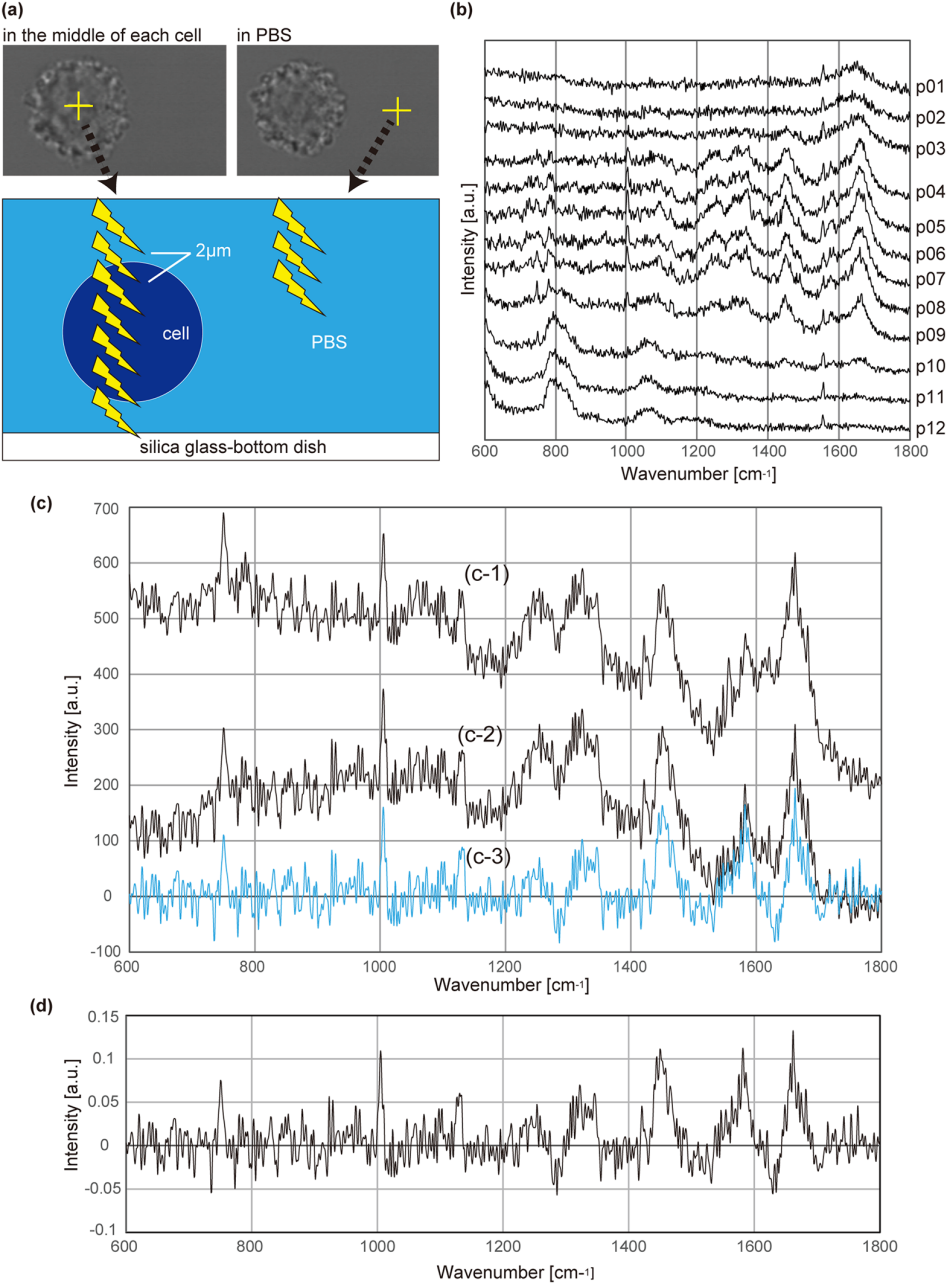
Acquisition of Raman spectra from cells. (**a**) Optical image of a KML1 cell. Cells were placed in silica glass-bottom dishes with phosphate-buffered saline (PBS). To evaluate each cell, Raman spectra were recorded in the middle of the cell, along the Z axis, at 2-μm intervals. To collect background signals, three Raman spectra also were recorded in the PBS next to each cell. (**b**) Representative multiple spectra obtained from one KML1 cell along the Z axis. Signals from inside the cell can be identified by the presence of various peaks in the fingerprint region. In the case presented in the panel, Spectra p04 to p09 corresponded to the signal from the cell and were used for further analyses. X-axis shows wavenumber [cm^−1^] and Y-axis shows signal intensity [a.u.: arbitrary unit]. (**c**, **d**) Method of data correction of the raw spectrum (**c-1**). The mean of 3 neighbouring spectra from PBS was subtracted from the raw spectrum (**c-2**). Baseline correction was performed using the I-ModPoly algorithm (**c-3**). To normalise the variation among data obtained on different days, signal intensity was corrected by vector normalisation (**d**). X-axis shows wavenumber [cm^−1^] and Y-axis shows signal intensity [a.u.: arbitrary unit].

**Figure 3:**
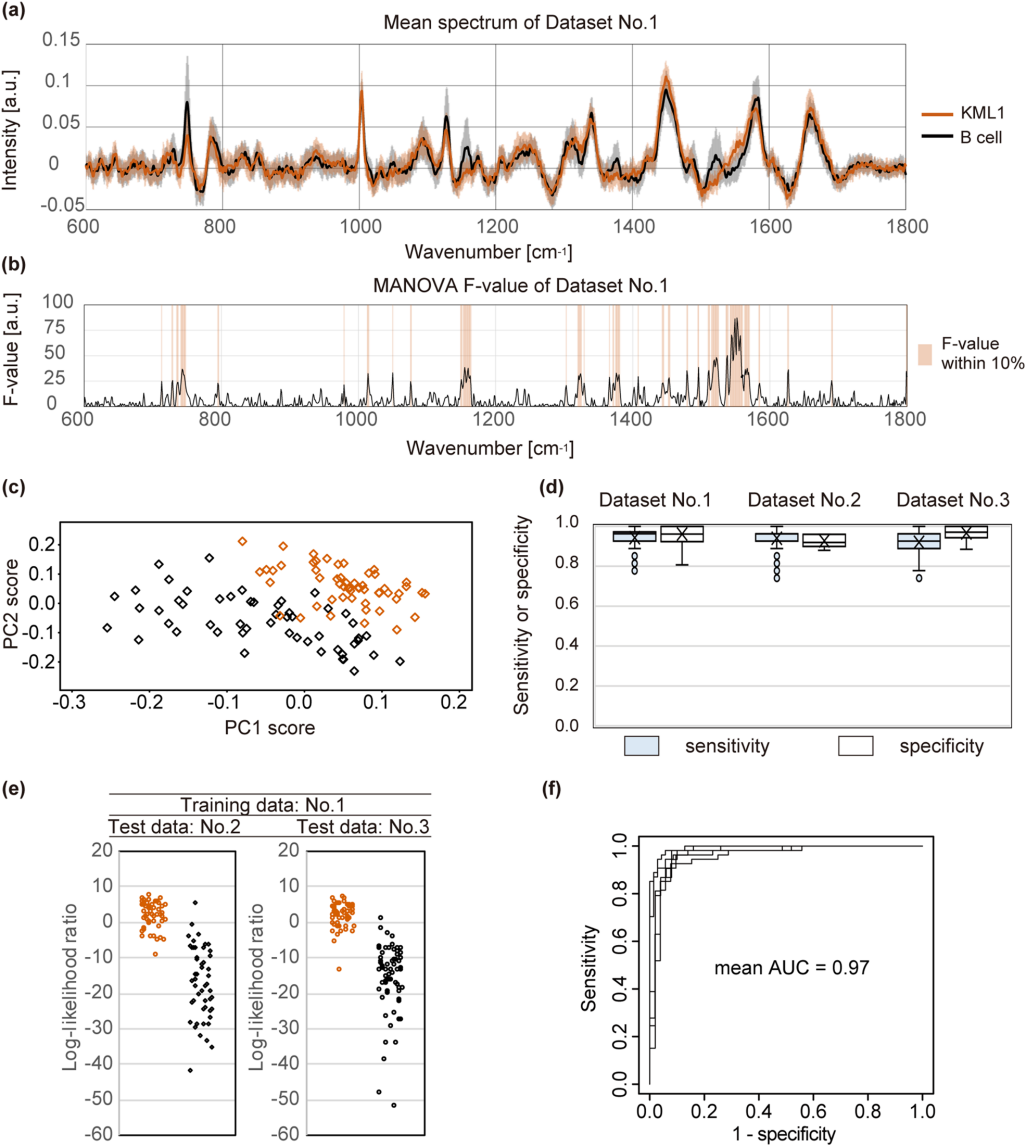
Comparison of spectra between B cells and KML1 cells. (**a**) Mean spectra of B cells (black) and KML1 cells (orange) in Dataset No. 1. The shaded regions indicate ± one standard deviation from the mean. X-axis shows wavenumber [cm^−1^] and Y-axis shows signal intensity [a.u.: arbitrary unit]. (**b**) F-values of multivariate analysis of variance (MANOVA) between two groups in Dataset No. 1. The shaded regions indicate wavenumbers with high F-values in the top 10% quantile. X-axis shows wavenumber [cm^−1^] and Y-axis shows F-value [a.u.: arbitrary unit]. (**c**) Scatter plot of principal component analysis component 1 (PC1) scores versus component 2 (PC2) scores of spectra in Dataset No. 1. Fifty-two spectra of B cells (black) and 54 spectra of KML1 cells (orange) were well clustered. X-axis shows PC1 score and Y-axis shows PC2 score. (**d**) Box and whisker plots of the sensitivity and specificity discriminating KML1 cells from B cells. Plots summarise 100 repeated intra-dataset evaluations in Datasets No. 1, No. 2, and No. 3. Boxes and horizontal lines indicate upper quartile, median, and lower quartile, respectively. X represents the mean value. Whiskers represent 1.5-times interquartile range from the upper and lower quartiles. Circles indicate outliers. (**e**) Scatter plot of log-likelihood ratio discriminating KML1 cells from B cells in inter-dataset analyses. The ratios were calculated by principal component analysis and quadratic discriminant analysis. (**f**) Receiver operating characteristic (ROC) curves were calculated for inter-dataset analyses. Mean value of the area under the ROC curves (AUC) was 0.97. X-axis shows 1-specificity and Y-axis shows sensitivity.

First, we compared the malignant lymphoma cell line KML1 to peripheral B cells, because in most intraocular lymphoma cases, lymphoma cells are derived from B cells. To evaluate each cell, Raman spectra were recorded in the middle of the cell, along the Z axis, at 2-μm intervals. To collect background signals, three Raman spectra also were recorded in the phosphate-buffered saline (PBS) next to each cell (Fig. 2a). Figure 2b shows representative multiple spectra obtained from one cell along the Z axis. Signals from inside the cells were identified by the presence of various peaks in the fingerprint region, and were used for further analyses (in this representative case, p04 to p09 were used). Fifty-two spectra from 12 B cells of Volunteer A and 54 spectra from 8 KML1 cells were evaluated in the first experiment (Dataset No. 1). Spectra were corrected by subtracting PBS signals, baseline correction, and vector normalisation, as described in the Materials and Methods section (Fig. 2c,d). The mean spectra of each cell type are shown in Fig. 3a. F-values of multivariate analysis of variance (MANOVA) between the two groups also were calculated to estimate variance between lymphoma and B cells (Fig. 3b). Signal intensities differed between lymphoma and B cells in wavenumbers with relatively large F-values. We analysed the spectra using principal component analysis (PCA) to visualise the complexed data by reducing the dimension. By PCA analysis, the 52 spectra of B cells and 54 spectra of KML1 cells were well clustered when labelled with principal component (PC) 1 scores and PC2 scores (Fig. 3c). We triplicated the experiments using B cells from other volunteers (Volunteer B and Volunteer C) on different days, yielding 3 datasets altogether (Datasets No. 1, No. 2, and No. 3). Cell numbers and spectra numbers in each experiment are listed in Supplementary Table S1. The mean spectra and F-values of Datasets No. 2 and No. 3 also were calculated (Supplementary Fig. S1). As in Dataset No. 1, differences in signal intensity between the two types of cells in Datasets No. 2 and No. 3 were obvious in wavenumbers with relatively large F-values. Such characteristic wavenumbers were reproducible in the triplicated datasets, and frequently were seen between 1400 cm^−1^ and 1600 cm^−1^. Signal intensities in the characteristic wavenumbers were not always large.

To assess the discriminating ability of Raman spectroscopy, we used the 3 datasets to perform intra-dataset analyses and inter-dataset analyses as described in the Materials and Methods. For intra-dataset analysis, 50% of the spectra were randomly selected as training data, and the remaining 50% were used as test data; the random selection was repeated 100 times. Classification models were built using PCA and quadratic discriminant analysis (QDA)^22^. Likelihood ratios of QDA to discriminate KML1 cells from B cells were calculated, and log-likelihood ratio = 0 was used as threshold value of the discrimination. Figure 3d plots the sensitivity and specificity for the discrimination of KML1 from B cells determined from 100 repeated evaluations. Collectively, the mean sensitivity was 0.94 and the mean specificity was 0.96 across the triplicate experiments. (Within the individual datasets, the respective sensitivities and specificities (mean ± standard deviation) were 0.94 ± 0.05 and 0.96 ± 0.04 in Dataset No. 1, 0.94 ± 0.05 and 0.94 ± 0.04 in Dataset No. 2, and 0.92 ± 0.06 and 0.97 ± 0.03 in Dataset No. 3.) These values varied when the discrimination threshold was changed, as shown graphically by Receiver Operating Characteristic (ROC) curves (Supplementary Fig. S2a). Mean ROC curves showed that specificities were high when sensitivities were between 0.95 and 0.99 in triplicate evaluations. The area under the ROC curve (AUC) represents the accuracy of the diagnostic methods; ROC-AUC = 0.50 indicates no discrimination, and ROC-AUC = 1.0 indicates perfect discrimination. Values of ROC-AUC between 0.50 and 0.70 represent low accuracy, between 0.70 and 0.90 represent moderate accuracy, and higher values represent high accuracy^23^. The mean ROC-AUC calculated from repeated intra-dataset analyses was 0.97 (0.97, 0.96, and 0.98 in Datasets No. 1–3, respectively; Supplementary Fig. S2a), showing the high diagnostic accuracy of Raman spectroscopy in discrimination of lymphoma cells from B cells.

Raman signals obtained in different experiments may have varied because the intensity of Raman peaks was influenced by any of multiple factors, including temperature and cell condition (e.g., due to donor differences). Therefore, we performed inter-dataset analyses to further assess the reproducibility of our results. Datasets No. 1–3 were used alternately as the training data or test data in 6 combinations. When Dataset No. 1 was used as the training data, the scatter plot of log-likelihood ratios for the spectra of Dataset No. 2 and for those of No. 3 showed similar distributions (Fig. 3e). This observation was confirmed by inter-dataset analyses using Datasets No. 2 and No. 3 (separately) as training datasets (Supplementary Fig. S2b). The sensitivities and specificities calculated by each of the 6 combinations of training data and test data are summarised in Supplementary Table S2. The mean sensitivity was 0.85 (range 0.74–0.94) and the mean specificity was 0.95 (range 0.90–1.00). The ROC-AUC value calculated from the 6 inter-dataset analyses ranged between 0.94 and 0.98 (mean 0.97, Fig. 3f), indicating reproducibility of the high diagnostic accuracy. These results showed the reproducibility of our analysis regardless of the variation due to day-to-day differences and donor differences, and confirmed the high discriminating ability of this technique.

### Discrimination of lymphoma cells from leukocytes involved in intraocular inflammatory diseases

**Figure 4:**
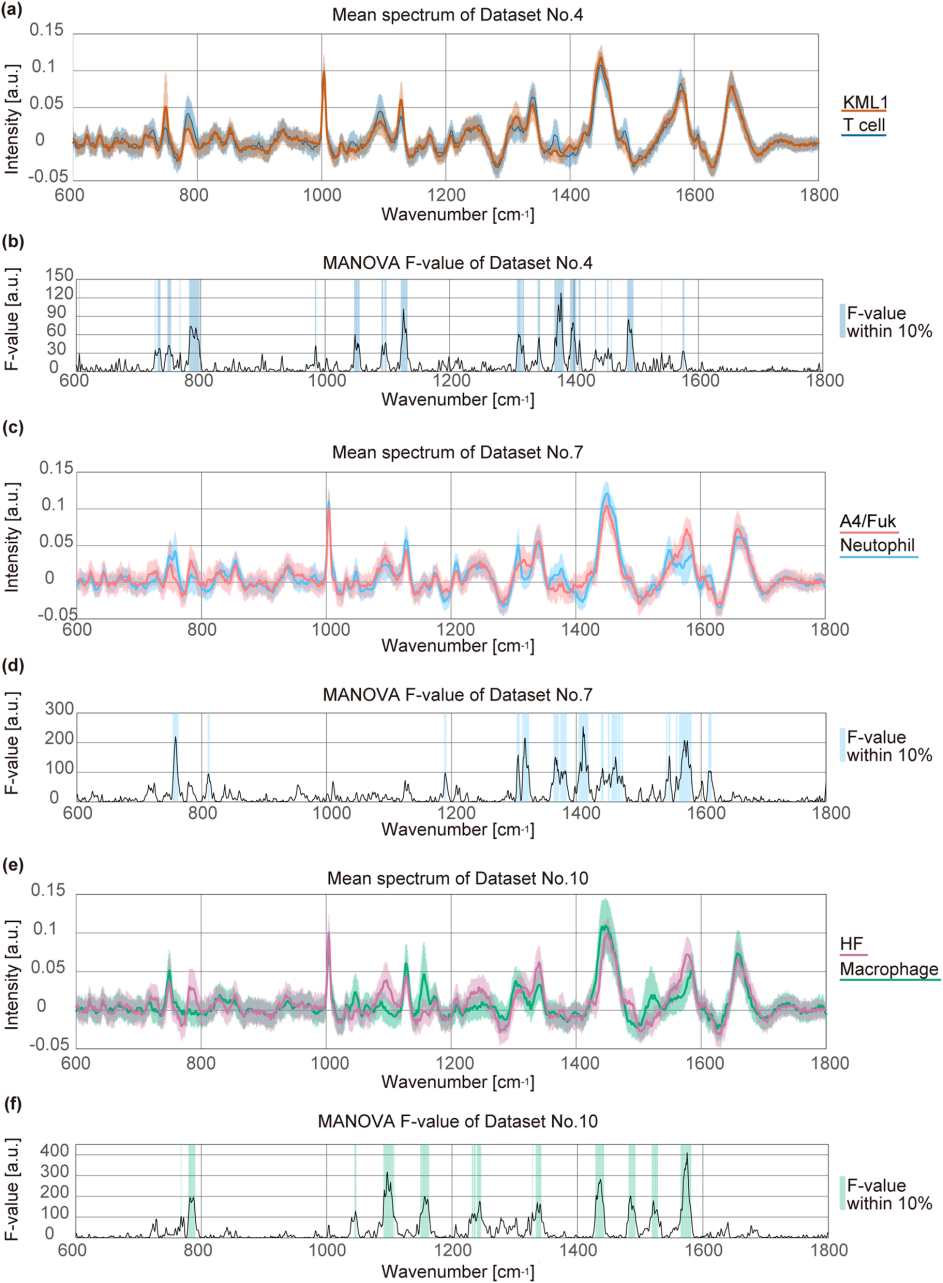
Mean spectra and F-values of lymphoma cells and inflammatory cells. (**a**) Mean spectra of KML1 cells (orange) and activated T cells (blue) in Dataset No. 4. The shaded regions indicate ± one standard deviation from the mean. (**b**) F-values of multivariate analysis of variance (MANOVA) between the two groups in Dataset No. 4. The shaded regions indicate wavenumbers with high F-values in the top 10% quantile. (**c**) Mean spectra of A4/Fuk cells (pink) and neutrophils (light blue) in Dataset No. 7. The shaded regions indicate ± one standard deviation from the mean. (**d**) F-values of MANOVA between the two groups in Dataset No. 7. The shaded regions indicate wavenumbers with high F-values in the top 10% quantile. (**e**) Mean spectra of HF cells (purple) and macrophages (green) in Dataset No. 10. The shaded regions indicate ± one standard deviation from the mean. (**f**) F-values of MANOVA between the two groups in Dataset No. 10. The shaded regions indicate wavenumbers with high F-values in the top 10% quantile. X-axis shows wavenumber [cm^−1^] and Y-axis shows signal intensity or F-value [a.u.: arbitrary unit].

As a second step, we proceeded to analyses that model the clinical situation that ophthalmologists face in differentiating intraocular lymphoma from intraocular inflammatory diseases. Most intraocular malignant lymphomas are classified as diffuse large B-cell lymphomas (DLBCLs). Since DLBCL is a heterogeneous disease, we used 3 distinct patient-derived cell lines (KML1, A4/Fuk, and HF). Activated T cells, neutrophils, and activated macrophages were used as control cells, because such cells play important roles in various diseases, including sarcoidosis, Behçet's disease, and bacterial endophthalmitis^24, 25^. We evaluated the model in triplicate using leukocytes from 3 donors (Volunteer A, B, and C) evaluated on different days, yielding 9 datasets altogether (activated T cells vs. KML1, Datasets No. 4–6; neutrophils vs. A4/Fuk, Datasets No. 7–9; and activated macrophages vs. HF, Datasets No. 10–12). The sample numbers are listed in Supplementary Tables S3-5. The mean spectra and MANOVA F-values of inflammatory cells obtained from Volunteer A and lymphoma cells are shown in Fig. 4 (Datasets No. 4, 7, 10). As in the comparison between B cells and KML1 cells, the signal intensities differed between control cells and lymphoma cells for wavenumbers with relatively large F-values. However, the distribution of such characteristic wavenumbers could be distinguished based on the type of control cells. The wavenumbers frequently were seen between 600 cm^−1^ and 1400 cm^−1^ when lymphoma cells were compared with activated T cells (Fig. 4a,b). However, the wavenumbers were seen between 1200 cm^−1^ and 1800 cm^−1^ when comparing between lymphoma and neutrophils (Fig. 4c,d), and were seen throughout the fingerprint region when comparing between lymphoma cells and macrophages (Fig. 4e,f). Reproducibility of these characteristic wavenumbers was confirmed using samples obtained from Volunteers B and C (Datasets No. 5, 6, 8, 9, 11 and 12; Supplementary Fig. S3).

**Figure 5:**
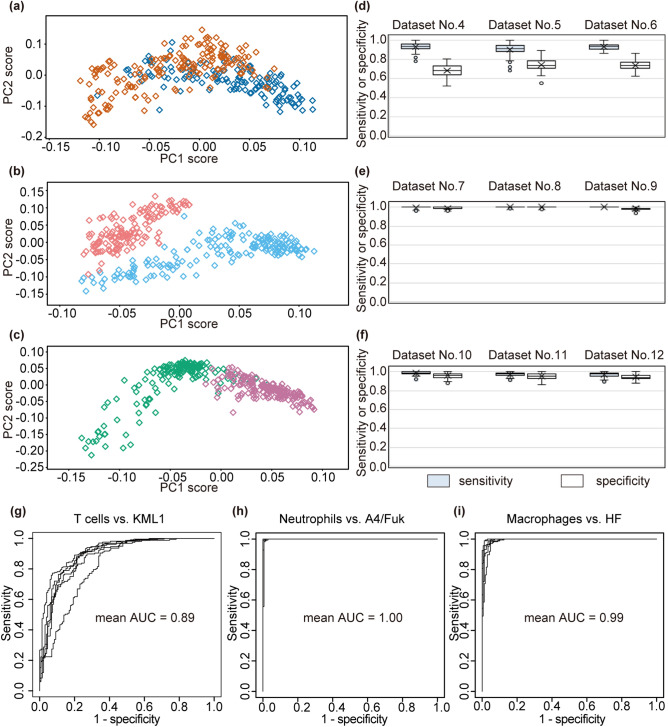
Comparison of spectra between lymphoma cells and inflammatory cells. (**a**–**c**) Scatter plot of principal component analysis component 1 (PC1) scores versus component 2 (PC2) scores of spectra in Dataset No. 4 (**a**), No. 7 (**b**), and No. 10 (**c**). One hundred and thirty-three spectra of T cells (blue), 167 spectra of KML1 cells (orange), 172 spectra of neutrophils (light blue), 146 spectra of A4/Fuk cells (pink), 168 spectra of macrophages (green), and 172 spectra of HF cells (purple) are shown. X-axis shows PC1 score and Y-axis shows PC2 score. (**d**–**f**) Box and whisker plots of the sensitivity and specificity discriminating KML1 cells from T cells (**d**), A4/Fuk cells from neutrophils (**e**), and HF cells from macrophages (**f**). Plots summarise 100 repeated intra-dataset evaluations in Datasets No. 4–12. Boxes and horizontal lines indicate upper quartile and median and lower quartile, respectively. X represents the mean value. Whiskers represent 1.5-times interquartile range from the upper and lower quartiles. Circles indicate outliers. Collectively, the respective mean sensitivity the mean specificity across the triplicate experiments were 0.92 and 0.72 (T cells vs. KML1 cells), 1.00 and 0.99 (neutrophils vs. A4/Fuk cells), and 0.97 and 0.95 (activated macrophages vs. HF cells). (**g**–**i**) Receiver operating characteristic (ROC) curves were calculated for inter-dataset analyses. Six ROC curves are shown in each graph. Although there was one outlier, the other 5 curves were reproducible in the comparison between T cells and KML1 cells (**g**). ROC curves for neutrophils and A4/Fuk (**h**), and for macrophages and HF cells (**i**) showed that the discriminating ability was reproducibly high. Mean values of the areas under the ROC curves (AUCs) were 0.89 (**g**), 1.00 (**h**), and 0.99 (**i**). X-axis shows 1-specificity and Y-axis shows sensitivity.

PCA analysis suggested smaller separation between the spectra of activated T cells and those of KML1 cells (Fig. 5a) compared to PCA analysis using neutrophils and macrophages (Fig. 5b,c). Next, we evaluated the discriminating ability by intra-dataset analyses and inter-dataset analyses. Figure 5d–f plots the sensitivity and specificity obtained by 100 repeated intra-dataset analyses. As shown by PCA plot, discrimination between activated T cells and KML1 (Fig. 5d) was more difficult than that between neutrophils and A4/Fuk (Fig. 5e) and that between macrophages and HF (Fig. 5f). Nonetheless, the discriminating ability between T cells and KML1 was reasonably high; mean sensitivity was 0.92 and specificity was 0.72, as calculated from the triplicate datasets. (Within the individual datasets, the respective sensitivities and specificities (mean ± standard deviation) were 0.93 ± 0.04 and 0.69 ± 0.06 in Dataset No. 4, 0.90 ± 0.06 and 0.74 ± 0.06 in Dataset No. 5, and 0.93 ± 0.03 and 0.74 ± 0.05 in Dataset No. 6.) For diagnostic use, sensitivity is more important than specificity because the false-negative rate should be reduced. ROC curves showed that specificities were 0.65, 0.69, and 0.70 (Datasets No. 4–6, respectively) when sensitivity was 0.95, indicating acceptable sensitivities and specificities for clinical application (Supplementary Fig. S4). Additionally, the mean ROC-AUC was determined to be 0.91 (0.90, 0.91, and 0.93 in Datasets No. 4–6, respectively), indicating that the analyses were categorised as a high-accuracy method^23^.

We also performed inter-dataset analyses to confirm the reproducibility of our data. Similar to the comparison between KML1 and B cells, each of the 3 datasets was alternately used as training data or test data. The sensitivities and specificities calculated by the 6 combinations of training data and test data are listed in Supplementary Tables S6-8. The mean sensitivity and specificity were 0.88 and 0.74 (T cells vs. KML1 cells), 1.00 and 0.99 (neutrophils vs. A4/Fuk cells), and 0.98 and 0.96 (activated macrophages vs. HF cells), indicating the discriminating ability was not greatly changed between intra-dataset analyses and inter-dataset analyses. To visualise the reproducibility of the discriminating ability, ROC curves were calculated. Although there was one outlier, the other 5 curves showed reproducibility in the comparison between T cells and KML1 cells (Fig. 5g). ROC curves for neutrophils and A4/Fuk (Fig. 5h), and for macrophages and HF cells (Fig. 5i) showed that the discriminating ability was reproducibly high. The range of the ROC-AUC values was 0.82–0.92 (mean 0.89) for T cells versus KML1 cells, 0.99–1.00 (mean 1.00) for neutrophils versus A4/Fuk cells, and 0.98–0.99 (mean 0.99) for macrophages versus HF cells. These results indicated the reproducibility of the discriminating ability by Raman spectroscopy.

## Discussion

In this study, we tested whether the Raman scattering properties of malignant cells without surrounding tissues were sufficient for the differentiation of malignant lymphoma. Specifically, we compared the Raman spectra from dissociated lymphoma cells to those from inflammatory cells that play important roles in intraocular inflammatory diseases, and evaluated whether we could discriminate the malignant cells based on cell spectra. We found that the Raman spectra from dissociated cells without confounding tissues showed a high discriminating ability regardless of the variation due to day-to-day and donor differences.

As controls, we used B cells, activated T cells, neutrophils, and activated macrophages. Specifically, B cells were used to make a comparison between malignant cells and normal cells with similar genetic backgrounds^26^, while activated T cells, neutrophils and activated macrophages were employed to make a comparison between lymphoma and inflammatory diseases. The latter analyses mimic the clinical situation in which ophthalmologists need to differentiate these diseases in the eye. Wavenumbers with high MANOVA F-values that would contribute to the discrimination differed for each of the four comparisons (Figs. 3a, b, 4, Supplementary Fig. S1 and S3). Raman peaks of cells are derived from various cellular molecules. For instance, the region between 800 cm^−1^ and 1200 cm^−1^ is reported to contain Raman peaks from nucleic acids, lipids (C–C and C–O stretching), proteins (C–C and C–N stretching), and carbohydrates (C–O stretching). On the other hand, the region between 1400 cm^−1^ and 1600 cm^−1^ is reported to contain peaks due to CH, CH_2_, and CH_3_ vibrations, and from C=C bonds (proteins)^27, 28^. It is not easy to clearly relate the changes in individual Raman peaks to variations in the metabolic and chemical status of cells, but the combinations of vibration of different molecules are presumed to contribute to the differentiation of lymphoma cells from each type of control cells.

In our multiple evaluations of the discriminating ability between lymphoma cells and normal B cells, sensitivity was 0.85–0.94 and specificity was 0.95–0.96 (Fig. 3d,f and Supplementary Table S2). The discriminating ability of Raman spectroscopy is affected by several factors, including microscope resolution, data processing method, and statistical analysis method. Although there is (to our knowledge) no previous report evaluating dissociated malignant lymphoma cells and normal B cells, Managò et al. compared dissociated B cell acute lymphoblastic leukaemia cells with normal B cells, obtaining a sensitivity of 0.88–0.94 and a specificity of 0.85–090^26^. These data indicated that the discriminating abilities evaluated by our experimental and analytical conditions were similar to those of previous reports.

In our comparisons between lymphoma cells and three types of inflammatory cells, sensitivities ranged between 0.88 and 1.00, and specificities ranged between 0.72 and 0.99 (Fig. 5d–i and Supplementary Tables S6-8). ROC-AUC values also confirmed the high accuracy of the diagnosis under these experimental conditions (Fig. 5g–i, Supplementary Fig. S4). Lloyd et al*.* used lymph node biopsy samples that contained not only malignant cells but also stroma and surrounding cells^20^. In their study, the signal intensity in wavenumbers assigned to collagen (858 cm^−1^, 877 cm^−1^, 939 cm^−1^, 1001 cm^−1^, 1030 cm^−1^, 1205 cm^−1^, 1240 cm^−1^, 1400 cm^−1^, and 1670 cm^−1^) were increased in malignant samples and were considered to be important factors for successful differentiation. In our experiments, however, the wavenumbers assigned to collagen showed low F-values and did not contribute much to the discrimination between control leukocytes and malignant lymphoma cells. This difference from the results of Lloyd et al*.* is presumed to reflect the elimination of surrounding tissues in our experiment. Lloyd et al*.* reported that Raman spectroscopy has a sensitivity of 0.90, a specificity of 0.86, and a ROC-AUC of 0.94 in the diagnosis of malignant disease. Surprisingly, the discriminating ability of Raman spectroscopy in our study was comparable to that in the study of Lloyd et al., although we did not include additional signal changes from deteriorated stroma and surrounding cells due to tumour infiltration. Conventional intraocular evaluation technologies mainly detect secondary environmental changes due to tumour invasion, rather than the characteristics of the invading cells. This aspect has been one of the reasons for the limited utility of conventional technologies in lymphoma diagnosis, given the diverse environmental changes that occur in various patients. The results of the present study suggest that Raman spectroscopy could serve as a novel diagnostic method in intraocular lymphoma. Furthermore, the discriminating ability was comparable to that of conventional cytology-based techniques that require surgery. In previous studies, elevated interleukin (IL) -10 concentrations or high IL-10:IL-6 ratios in the intraocular fluid were found to have sensitivities of 0.74–0.89 and specificities of 0.75–0.93^15^. The technique described here, Raman spectroscopy, can be employed for in vivo evaluation without requiring surgery, and is expected to be useful for shortening the duration between the onset of symptoms and diagnosis. The limitation of this study is that the cells invading the diseased eye are very diverse. For example, granulomas, monocytes, eosinophils and melanin-containing epithelioid cells have been reported to be involved in the vitreous opacities observed with intraocular inflammatory diseases^24, 25^. Clearly, additional studies using various control cells will be necessary to determine the utility of Raman spectroscopy in this context.

Several previous studies have reported the successful application of Raman effects for single-cell analysis or Raman cytometry^26, 29^. In ophthalmology, Raman spectroscopy has been used primarily for the evaluation of gross eye structures such as retina, lens, and cornea^13^. Our study is the first (to our knowledge) to suggest the potential utility of in vivo Raman cytometry in the human eye. However, several barriers will need to be overcome before Raman cytometry can be applied in clinical ophthalmological settings. First, the power of the laser will need to be weak enough to meet safety criteria for eye evaluation^30^. However, signal intensities in wavenumbers with high F-values were not always large (Figs. 3a, b, 4, Supplementary Fig. S1 and S3). Collecting the full spectrum, including small peaks, will be important for discrimination, as noted in a previous study using a different type of cells^22^. Laser power will need to meet these two requirements. Second, the laser exposure time will need to be shortened. In vivo, cells in the eye may move due to thermal differences between the temperature of body and outside environment. In this report, we analysed spectra regardless of cell region (nucleus and cytosol) because we thought it would be difficult to evaluate the Raman spectra of specific cell compartments in vivo. Some researchers have made efforts to increase the speed of Raman detection^31, 32^. Shortening of the exposure time may make the discrimination more accurate by permitting measurement of defined regions within cells. In addition, given that the Raman spectroscope is an expensive, high-precision machine, cost may be a challenge for the widespread use of Raman spectroscopy in clinical applications. Finally, the discrimination threshold value should be optimised. As shown by ROC curves, sensitivity and specificity varied continuously as the threshold values changed. After in vivo application is realised technically, it is expected that large amounts of data will be obtained, including Raman spectra along with evaluations by conventional cytology-based technologies. It is critical that false-negative rates are decreased in the diagnosis of malignant disease; threshold values with high sensitivity therefore will be desirable. The eye has a transparent structure suitable for optical observation. Raman spectroscopy does not require any labelling and is suitable for live imaging. When these barriers are overcome by interdisciplinary approaches, in vivo Raman cytometry in the eye is expected to contribute not only to saving patient lives but also to achieving novel understanding in various research fields, including cell biology, oncology, and immunology.

## Materials and methods

### Cell culture

KML1, a cell line established from the pleural effusion of a patient with diffuse large B cell lymphoma (DLBCL), and A4/Fuk, a cell line established from the ascites fluid of a patient with DLBCL, were purchased from the National Institutes of Biomedical Innovation, Health and Nutrition (JCRB1347 and JCRB0097, respectively). HF is another malignant lymphoma cell line, and was established from the pleural effusion of a patient with DLBCL; this line was purchased from American Type Culture Collection (CRL­3383). Cryopreserved lymphoma cell lines were thawed and cultured in RPMI 1640 supplemented with foetal bovine serum (FBS; 10% for KML1 and A4/Fuk, and 15% for HF) for 2 days before observation by Raman spectroscopy. As control cells, peripheral B cells, activated T cells, neutrophils, and activated macrophages were used. Peripheral B cells, T cells, neutrophils, and macrophages were isolated from peripheral blood of 3 healthy volunteers (Volunteers A, B, and C) using the EasySep^™^ Human Pan-B Cell Enrichment Kit (Veritas, ST-19554), the EasySep^™^ Direct Human T Cell Isolation Kit (Veritas, ST-19661), the EasySep^™^ Direct Human Neutrophil Isolation kit (Veritas, ST-19666), and the EasySep^™^ Human Monocyte Isolation Kit (Veritas, ST-19359). These procedures were performed in accordance with an ethical research proposal approved by Medical Research Ethics Committee, Tokyo Medical and Dental University (M2017-292). Written informed consent was obtained from each donor. All experiments were performed in accordance with the tenets of the Declaration of Helsinki. Isolated B cells were maintained in RPMI 1640 supplemented with 10% FBS, and these cells were evaluated by Raman spectroscopy on the day of isolation. Isolated T cells were further activated by 1-week culturing in RPMI 1640 supplemented with 10% FBS along with Dynabeads Human T-Activator CD3/CD28 (Veritas, DB11131) and IL-2 (Corning, 354,043) in a 37 °C, 5% CO_2_ atmosphere. Isolated macrophages were activated according to the manufacturer’s 6-day culture protocol using ImmunoCult™-SF Macrophage Differentiation Medium (STEMCELL, 10,961) supplemented with 50 ng/mL macrophage colony stimulating factor, 10 ng/mL lipopolysaccharides, and 50 ng/ mL interferon gamma. Lymphoma cell lines, B cells, T cells, and macrophages were transported to the confocal Raman microscope in the respective culture media using a live-transport device and constant-temperature transport box (Sanplatec, iP-TEC^®^). Since neutrophils are fragile, cells of this type were isolated at the place of observation and were evaluated promptly.

### Raman spectroscopy evaluation and pre-analysis data correction

Scanning was performed using a RAMANplus (Nanophoton Corp., Osaka, Japan) located at the National Institute for Materials Science, Japan. Cells were maintained in the CO_2_ incubator until observation. For Raman spectroscopy, cells were transferred to silica glass-bottom dishes (Matsunami Glass, D1130S) containing 1 mL PBS without Ca^2+^ and Mg^2+^ (PBS (−)). For each experiment, Raman spectra were collected at room temperature within 60 min using a 100 × water-immersion objective lens (Nikon, CFI Plan 100XC W, NA 1.10). The excitation source was a 523-nm laser. Laser power was 4.5 mW and the exposure time was 9 s, conditions that had been determined (in preliminary experiments; Supplementary Fig. S5) to be suitable for achieving good spectrum quality without tissue damage. We confirmed that the cells remained unbroken after evaluation. The central wavenumber was 1800 cm^−1^ and a diffraction grating of 600 gr mm^−1^ was used. The spectral resolution was 4 cm^−1^ and the spatial resolution was 500 nm. The Raman band of free nitrogen (2330 cm^−1^) was used for post calibration. Only the fingerprint region (600–1800 cm^−1^) was used for statistical analysis because various Raman peaks derived from biomolecules in cells can be observed in this region^27, 33^. To evaluate each cell, the Raman spectra were recorded across the middle of the cell, along the Z axis, at 2-μm intervals (Fig. 2a, b). Typically, scanning of a single cell required 5 to 10 Z-scan measurements, depending on the cell size. Thus, it took about 45 s to 1.5 min to evaluate each cell. To collect background signals, 3 Raman spectra also were recorded in the PBS (−) next to each cell, again along the Z axis at 2-μm intervals (Fig. 2a). Among multiple spectra obtained from each cell along the Z axis, signals from inside the cells were identified by the presence of various peaks in the fingerprint region (Fig. 2b). Figure 2c,d show how we corrected the raw spectra (Fig. 2c1). The mean of 3 neighbouring spectra (i.e., those recorded from PBS (-)) was subtracted from the raw spectra (Fig. 2c2). Baseline correction was performed using the I-ModPoly algorithm^34^ (Fig. 2c3). To normalise the variation among data obtained on different days, signal intensity was corrected by vector normalisation (Fig. 2d). All data correction and analysis were performed using R version 3.4.3 (2017-11-30)^35^.

### Multivariate analysis

To estimate the variance between lymphoma cells and control cells, F-values were calculated by MANOVA. Classification models were built using PCA and QDA. In the discriminant analysis, all principal components with statistically significant F-values (*p* < 0.05) were included, and the qda() function in the MASS package of R was used. For intra-dataset analyses, 50% of the spectra were randomly selected as training data, and the remaining 50% were used as test data. Random selection was repeated 100 times, and the sensitivity and specificity of the diagnosis were calculated. A receiver operating characteristic (ROC) curve also was calculated to evaluate the diagnostic accuracy of the analyses. For inter-dataset analyses, datasets obtained using different volunteers’ control cells on different day were used as the training dataset or test dataset.

## Conclusions

Raman spectra from dissociated cells without surrounding tissues showed high ability to discriminate lymphoma cells from leukocytes involved in intraocular inflammatory diseases. Raman spectroscopy may serve as an attractive tool for intraocular evaluation.

## Supplementary information

Supplementary Information 1.

Supplementary Information 2.

## Data Availability

Data and materials are available from the corresponding author upon reasonable request.

## References

[1] Lui H, Zhao J, McLean D, Zeng H (2012). Real-time Raman spectroscopy for in vivo skin cancer diagnosis. Cancer Res..

[2] Lim L (2014). Clinical study of noninvasive in vivo melanoma and nonmelanoma skin cancers using multimodal spectral diagnosis. J. Biomed. Opt..

[3] Zhang Y (2020). Assessment of Raman spectroscopy for reducing unnecessary biopsies for melanoma screening. Molecules.

[4] Jermyn M (2015). Intraoperative brain cancer detection with Raman spectroscopy in humans. Sci. Transl. Med..

[5] Lin D (2018). Autofluorescence and white light imaging-guided endoscopic Raman and diffuse reflectance spectroscopy for in vivo nasopharyngeal cancer detection. J. Biophotonics.

[6] Malik A (2017). In vivo Raman spectroscopy-assisted early identification of potential second primary/recurrences in oral cancers: an exploratory study. Head Neck.

[7] McGregor HC (2017). Real-time endoscopic Raman spectroscopy for in vivo early lung cancer detection. J. Biophotonics.

[8] Desroches J (2018). A new method using Raman spectroscopy for in vivo targeted brain cancer tissue biopsy. Sci. Rep..

[9] O'Brien CM (2018). In vivo Raman spectroscopy for biochemical monitoring of the human cervix throughout pregnancy. Am. J. Obstet. Gynecol..

[10] Enejder AM (2005). Raman spectroscopy for noninvasive glucose measurements. J. Biomed. Opt..

[11] Bernstein PS (2002). Resonance Raman measurement of macular carotenoids in normal subjects and in age-related macular degeneration patients. Ophthalmology.

[12] Stiebing C (2019). Nonresonant Raman spectroscopy of isolated human retina samples complying with laser safety regulations for in vivo measurements. Neurophotonics.

[13] Erckens RJ (2001). Raman Spectroscopy in ophthalmology: from experimental tool to applications in vivo. Lasers Med. Sci..

[14] Chan CC (2011). Primary vitreoretinal lymphoma: a report from an International Primary Central Nervous System Lymphoma Collaborative Group symposium. Oncologist.

[15] Sagoo MS (2014). Primary intraocular lymphoma. Surv. Ophthalmol..

[16] Grimm SA (2007). Primary intraocular lymphoma: an international primary central nervous system lymphoma collaborative group report. Ann. Oncol..

[17] Davis JL, Viciana AL, Ruiz P (1997). Diagnosis of intraocular lymphoma by flow cytometry. Am. J. Ophthalmol..

[18] Lobo A, Lightman S (2003). Vitreous aspiration needle tap in the diagnosis of intraocular inflammation. Ophthalmology.

[19] Donfack P, Grote K, Lerchl A, Materny A (2013). Probing lymphoma infiltration in spleen of AKR/J mice chronically exposed to electromagnetic fields for risk assessment–toward noninvasive modeling. J. Biophotonics.

[20] Lloyd GR (2013). Discrimination between benign, primary and secondary malignancies in lymph nodes from the head and neck utilising Raman spectroscopy and multivariate analysis. Analyst.

[21] Rau JV (2019). Raman spectroscopy discriminates malignant follicular lymphoma from benign follicular hyperplasia and from tumour metastasis. Talanta.

[22] Germond A (2018). Raman spectral signature reflects transcriptomic features of antibiotic resistance in Escherichia coli. Commun. Biol..

[23] Swets JA (1988). Measuring the accuracy of diagnostic systems. Science.

[24] Coupland SE (2008). The pathologist's perspective on vitreous opacities. Eye (Lond.).

[25] Matsuo T, Ichimura K (2012). Immunocytochemical diagnosis as inflammation by vitrectomy cell blocks in patients with vitreous opacity. Ophthalmology.

[26] Managò S (2016). A reliable Raman-spectroscopy-based approach for diagnosis, classification and follow-up of B-cell acute lymphoblastic leukemia. Sci. Rep..

[27] Movasaghi Z, Rehman S, Rehman IU (2007). Raman spectroscopy of biological tissues. Appl. Spectrosc. Rev..

[28] Auner GW (2018). Applications of Raman spectroscopy in cancer diagnosis. Cancer Metastasis Rev..

[29] Li M, Xu J, Romero-Gonzalez M, Banwart SA, Huang WE (2012). Single cell Raman spectroscopy for cell sorting and imaging. Curr. Opin. Biotechnol..

[30] Landry RJ, Bostrom RG, Miller SA, Shi D, Sliney DH (2011). Retinal phototoxicity: a review of standard methodology for evaluating retinal optical radiation hazards. Health Phys..

[31] Camp CH, Yegnanarayanan S, Eftekhar AA, Adibi A (2011). Label-free flow cytometry using multiplex coherent anti-Stokes Raman scattering (MCARS) for the analysis of biological specimens. Opt. Lett..

[32] Hiramatsu K (2019). High-throughput label-free molecular fingerprinting flow cytometry. Sci. Adv..

[33] De Gelder J, De Gussem K, Vandenabeele P, Moens L (2007). Reference database of Raman spectra of biological molecules. J. Raman Spectrosc..

[34] Zhao J, Lui H, McLean DI, Zeng H (2007). Automated autofluorescence background subtraction algorithm for biomedical Raman spectroscopy. Appl. Spectrosc..

[35] R Core Team. R: A language and environment for statistical computing. R Foundation for Statistical Computing, Vienna, Austria. URL https://www.R-project.org/. (2017).

