# Supplementary material for: Discrimination of dissociated lymphoma cells from leukocytes by Raman spectroscopy
Source: Sci Rep. 2020 Sep 25;10:15778. doi: 10.1038/s41598-020-72762-5 (PMC7519070; doi:10.1038/s41598-020-72762-5)
Supplement: Supplementary file 1 — Supplementary Information 1. [file 41598_2020_72762_MOESM1_ESM.pdf]

Discrimination of dissociated lymphoma cells from leukocytes by Raman spectroscopy

Yuko Iwasaki, Masahiko Kawagishi, Hiroshi Takase, and Kyoko Ohno-Matsui

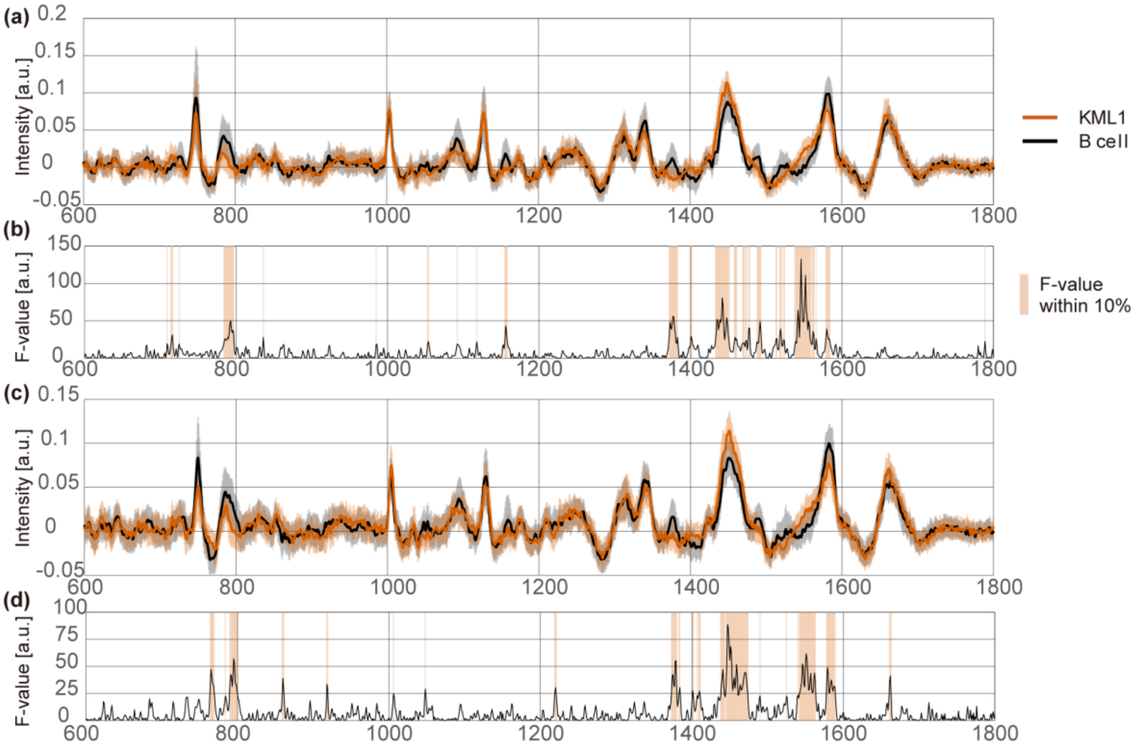

Supplementary Figure S1. Comparison of mean spectra and F-values from Datasets No. 2 and 3.

(a, c) Mean spectra of B cells (black) and KML1 cells (orange) in Datasets No. 2 (a) and No. 3 (c). The shaded regions indicate ± one standard deviation from the mean.

(b, d) F-values of multivariate analysis of variance (MANOVA) in Datasets No. 2 (b) and No. 3 (d). The shaded regions indicate wavenumbers with high F-values in the top 10% quantile. Note that high F-values frequently were seen between 1400 cm^{-1} and 1600 cm^{-1} for B cells. X-axis shows wavenumber [cm^{-1}] and Y-axis shows signal intensity or F-value [a.u.: arbitrary unit].

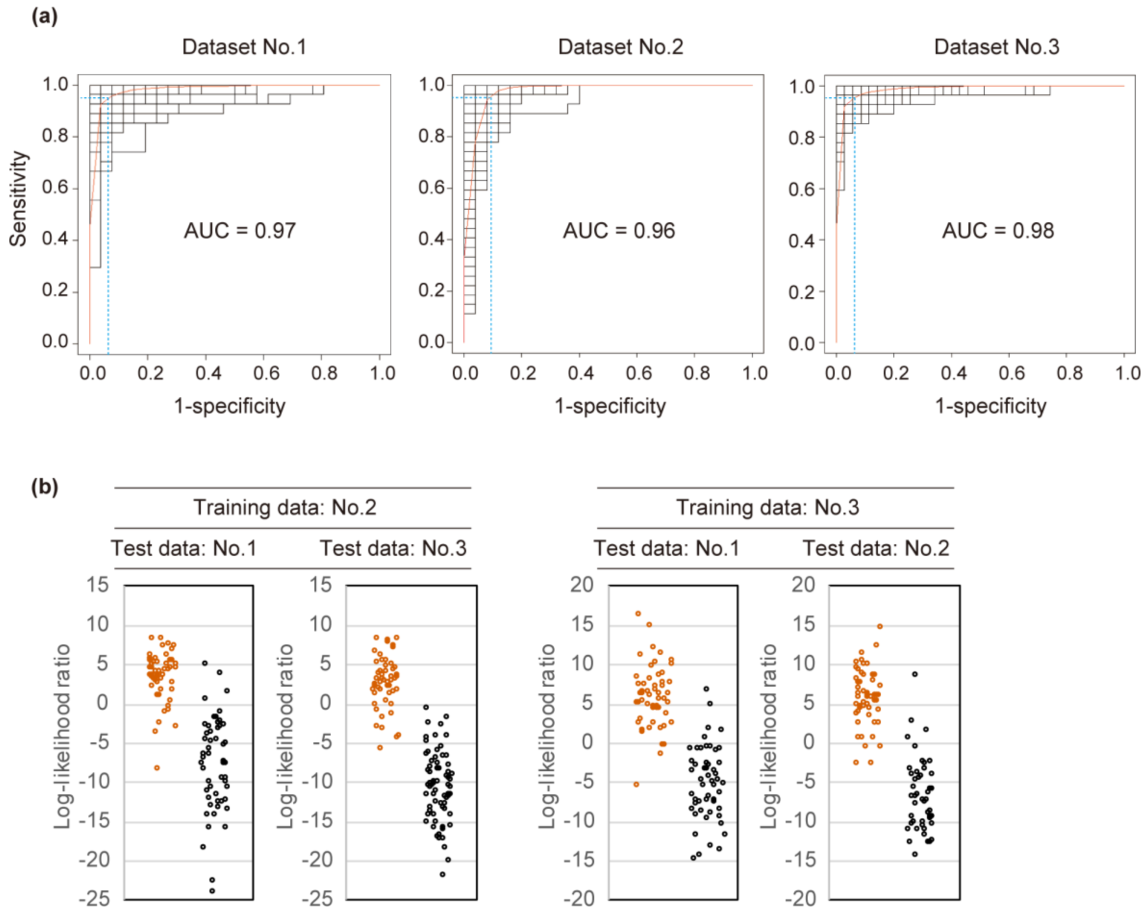

Supplementary Figure S2. Evaluation of the accuracy and robustness of the discrimination between lymphoma cells and B cells.

(a) Receiver operating characteristic (ROC) curves were calculated for intra-dataset analyses in Datasets No. 1, No. 2, and No. 3. Dotted blue lines indicate sensitivities of 0.95. Mean ROC curves showed that when discrimination thresholds were set such that sensitivities were 0.95, specificities were 0.93, 0.91, and 0.94 (Dataset No. 1, 2, and 3, respectively). In 100 repeated evaluations, the mean values of the areas under the ROC curves (AUCs) for each dataset were 0.97, 0.96, and 0.98, respectively. X-axis shows 1-specificity and Y-axis shows sensitivity.

(b) Scatter plot of log-likelihood ratio for discriminating KML1 cells from B cells in inter-dataset analyses. The ratios were calculated by principal component analysis and quadratic discriminant analysis.

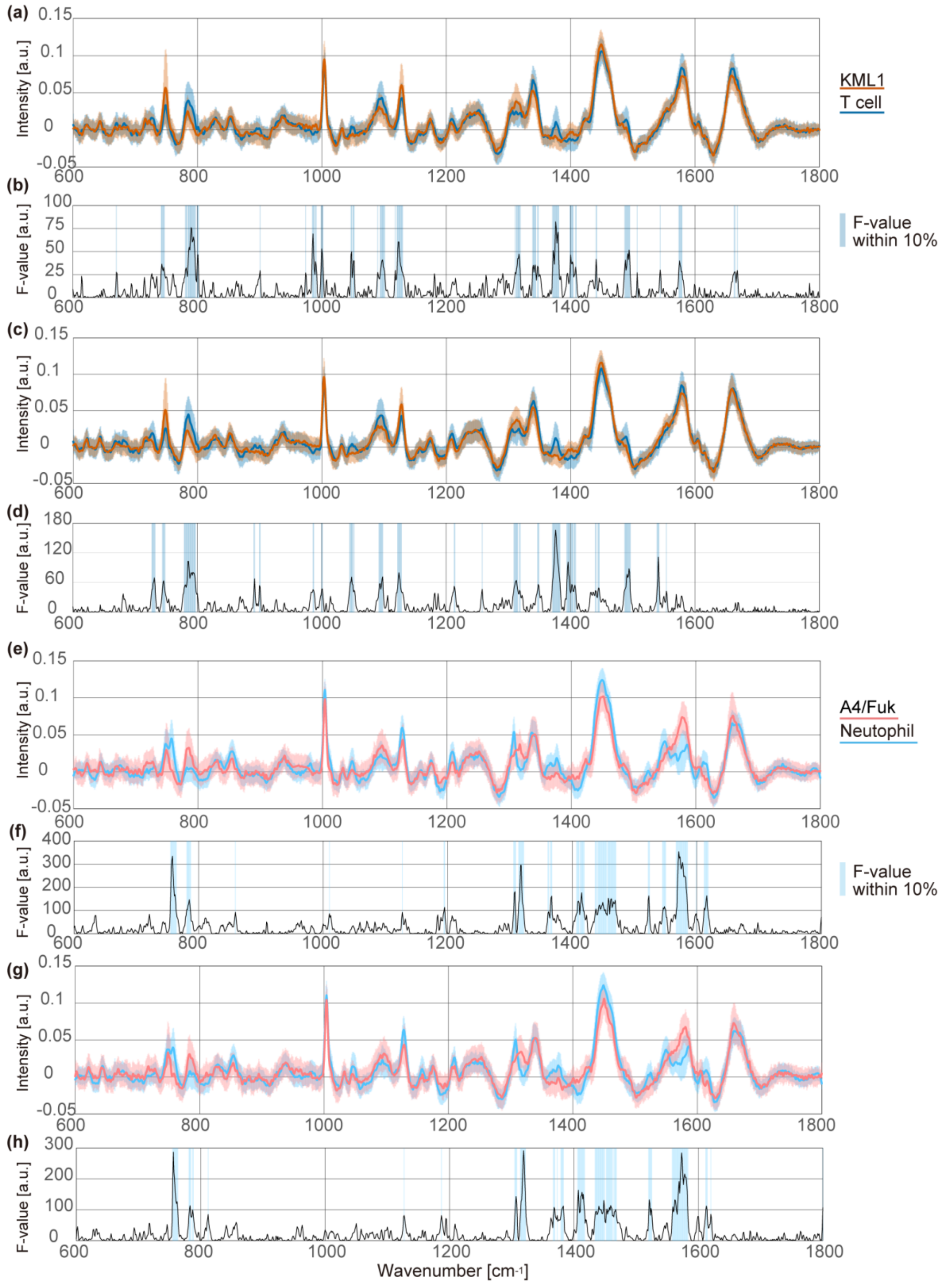
(a)
(b)
(c)
(d)
(e)
(f)
(g)
(h)
Intensity [a.u.]
F-value [a.u.]
KML1
T cell
F-value within 10%
A4/Fuk
Neutophil
Wavenumber [cm-1]

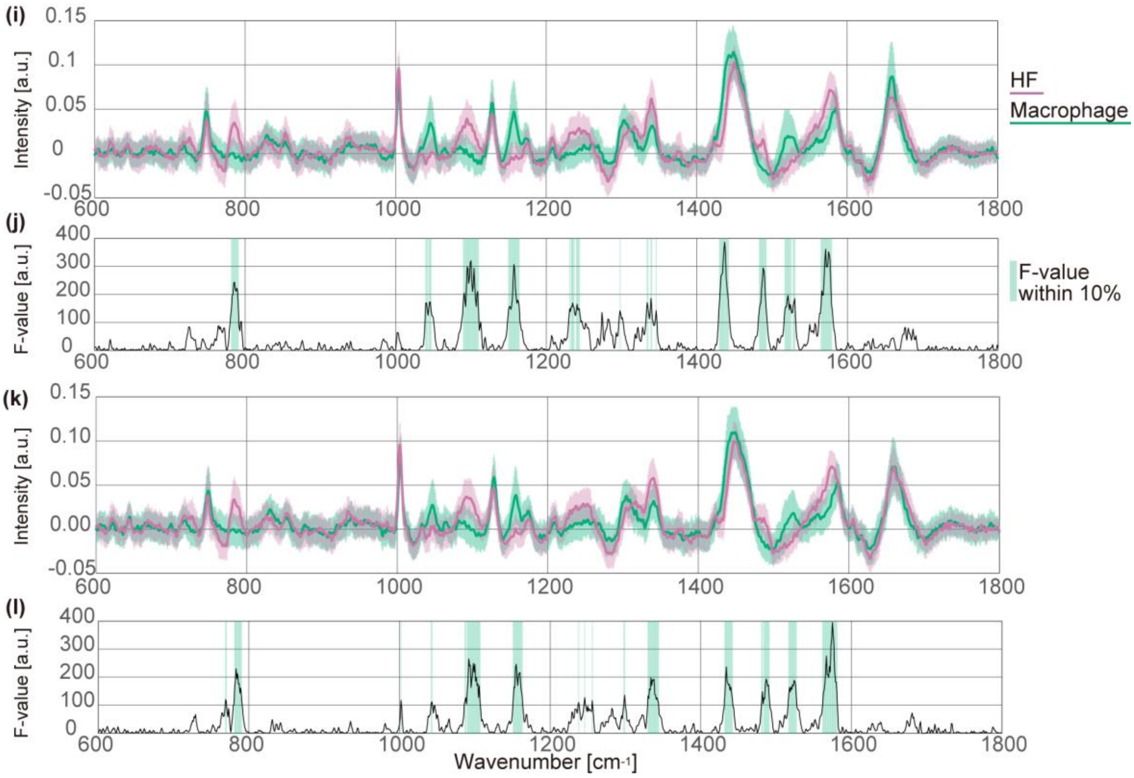

Supplementary Figure S3. Comparison of mean spectra and F-values from Datasets No. 5, 6, 8, 9, 11, and 12.

(a, c, e, g, i, k) Mean spectra of KML1 cells (orange) and T cells (blue) in Datasets No. 5 (a) and No. 6 (c), A4/Fuk cells (pink) and neutrophils (light blue) in Datasets No. 8 (e) and No. 9 (g), and HF cells (purple) and macrophages (green) in Datasets No. 11 (i) and No. 12 (k). The shaded regions indicate ± one standard deviation from the mean.

(b, d, f, h, j, l) F-values of multivariate analysis of variance (MANOVA) in Datasets No. 5 (b), No. 6 (d), No. 8 (f), No. 9 (h), No. 11 (j), and No. 12 (l). The shaded regions indicate wavenumbers with high F-values in the top 10% quantile. Note that high F-values frequently were seen between 600 cm^{-1} and 1400 cm^{-1} for T cells (b, d), between 1200 cm^{-1} and 1800 cm^{-1} for neutrophils (f, h), and throughout the fingerprint region for macrophages (j, l). X-axis shows wavenumber [cm^{-1}] and Y-axis shows signal intensity or F-value [a.u.: arbitrary unit].

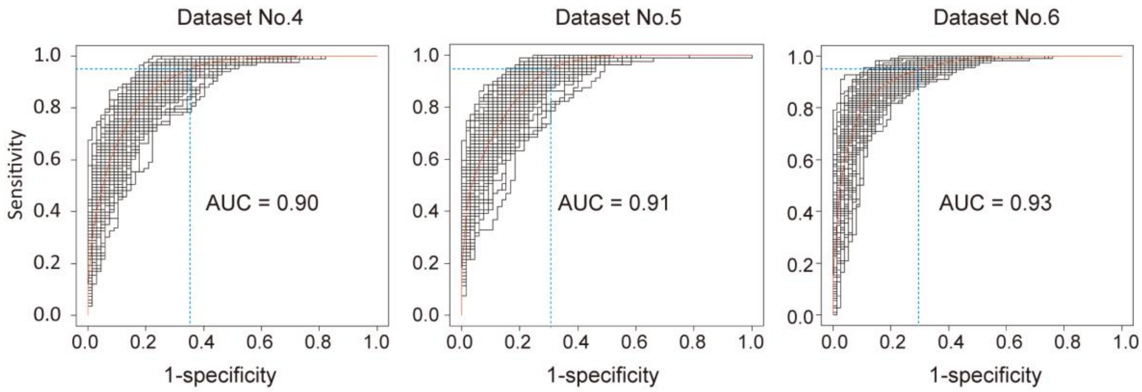

Supplementary Figure S4. Receiver operating characteristic curves calculated for intra-dataset analyses between lymphoma cells and T cells.

Receiver operating characteristic (ROC) curves were calculated for intra-dataset analyses in Datasets No. 4, No. 5, and No. 6. Dotted blue lines indicate sensitivities of 0.95. Mean ROC curves showed that when discrimination thresholds were set such that sensitivities were 0.95, specificities were 0.65, 0.69, and 0.70 (Dataset No. 4, 5, and 6, respectively). In 100 repeated evaluations, the mean values of the areas under the ROC curves (AUCs) for each dataset were 0.90, 0.91, and 0.93, respectively. X-axis shows 1-specificity and Y-axis shows sensitivity.

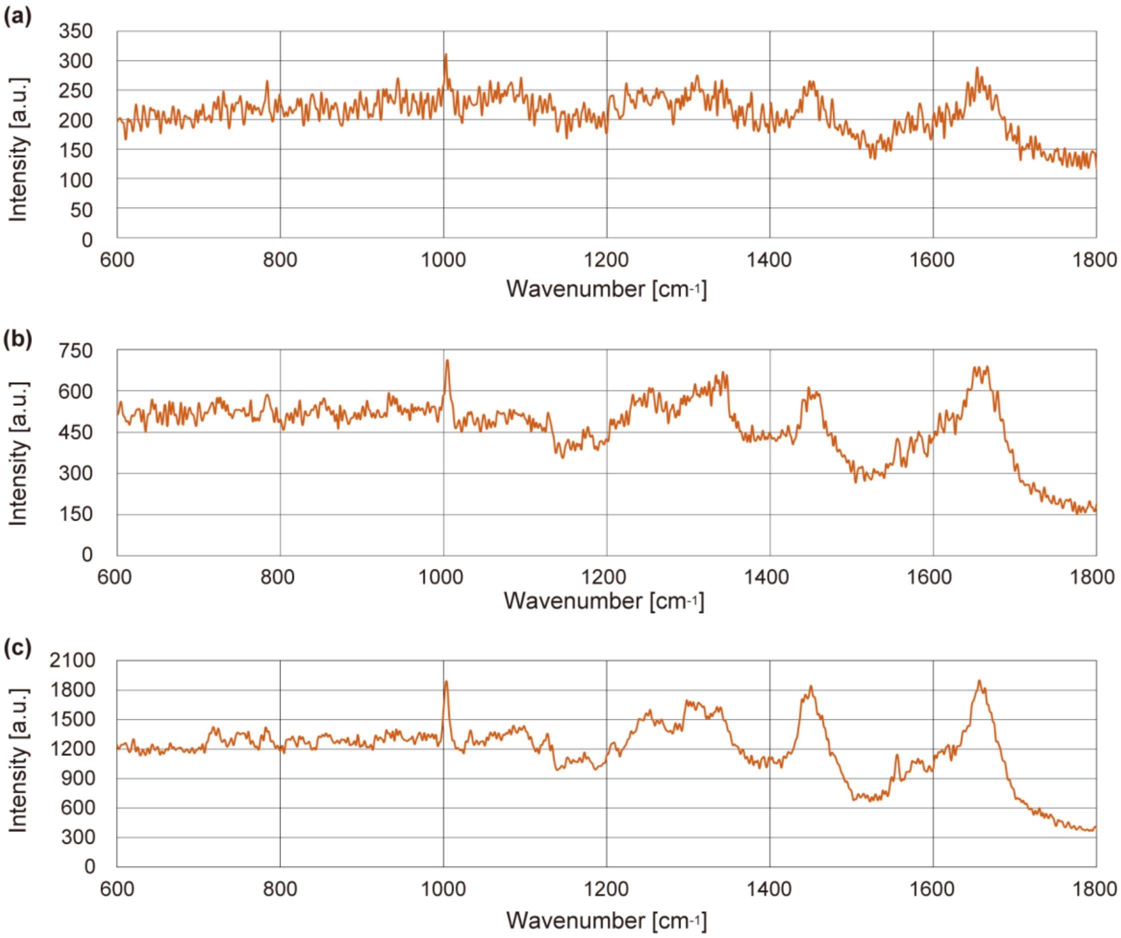

Supplementary Figure S5. Comparison of spectra from KML1 cells obtained using different laser powers.

(a) 1 mW, (b) 4.5 mW, and (c) 9 mW. The exposure duration was 9 sec in each of the 3 evaluations. Spectra obtained with laser at 4.5 mW and 9 mW showed improved signal-to-background ratio compared to that obtained with laser at 1 mW. To avoid possible tissue damage, we used the laser at 4.5 mW for all experiments. X-axis shows wavenumber [cm^{-1}] and Y-axis shows signal intensity [a.u.: arbitrary unit].
