# Supplementary material for: Discrimination of dissociated lymphoma cells from leukocytes by Raman spectroscopy
Source: Sci Rep. 2020 Sep 25;10:15778. doi: 10.1038/s41598-020-72762-5 (PMC7519070; doi:10.1038/s41598-020-72762-5)
Supplement: Supplementary file 2 — Supplementary Information 2. [file 41598_2020_72762_MOESM2_ESM.pdf]

Discrimination of dissociated lymphoma cells from leukocytes by Raman spectroscopy

Yuko Iwasaki, Masahiko Kawagishi, Hiroshi Takase, and Kyoko Ohno-Matsui

Table S1. Sample numbers in comparison between B cells and KML1

	Cell type	Dataset		
		No. 1	No. 2	No. 3
Cell number (Spectrum number)	B cell	12(52)	12 (50)	15 (70)
	KML1	8(54)	8 (53)	8 (54)

Table S2. Discrimination ability between B cells and KML1 evaluated by 6 inter-dataset analyses

Dataset		Sensitivity	Specificity
Training	Test		
No. 1	No. 2	0.77	0.98
	No. 3	0.74	0.99
No. 2	No. 1	0.87	0.92
	No. 3	0.85	1.00
No. 3	No. 1	0.94	0.90
	No. 2	0.92	0.92
Mean value		0.85	0.95

Table S3. Sample numbers in comparison between T cells and KML1

	Cell type	Dataset		
		No. 4	No. 5	No. 6
Cell number (Spectrum number)	T cell	32 (133)	31 (130)	37 (150)
	KML1	25 (167)	24 (157)	32 (220)

Table S4. Sample numbers in comparison between neutrophils and A4/Fuk

	Cell type	Dataset		
		No. 7	No. 8	No. 9
Cell number (Spectrum number)	Neutrophil	39 (172)	36 (162)	36 (164)
	A4/Fuk	24 (146)	24 (155)	25 (167)

Table S5. Sample numbers in comparison between macrophages and HF

	Cell type	Dataset		
		No. 10	No. 11	No. 12
Cell number (Spectrum number)	Macrophage	24 (168)	25 (178)	28 (168)
	HF	25 (172)	25 (161)	26 (174)

Table S6. Discrimination ability between activated T cells and KML1 evaluated by 6 inter-dataset analyses

Dataset		Sensitivity	Specificity
Training	Test		
No. 4	No. 5	0.90	0.64
	No. 6	0.93	0.69
No. 5	No. 4	0.79	0.80
	No. 6	0.85	0.81
No. 6	No. 4	0.90	0.74
	No. 5	0.92	0.73
Mean value		0.88	0.74

Table S7. Discrimination ability between neutrophils and A4/Fuk evaluated by 6 inter-dataset analyses

Dataset		Sensitivity	Specificity
Training	Test		
No. 7	No. 8	1.00	1.00
	No. 9	1.00	0.99
No. 8	No. 7	1.00	0.97
	No. 9	1.00	0.98
No. 9	No. 7	0.99	0.99
	No. 8	1.00	1.00
Mean value		1.00	0.99

Table S8. Discrimination ability between activated macrophages and HF evaluated by 6 inter-dataset analyses

Dataset		Sensitivity	Specificity
Training	Test		
No. 10	No. 11	0.96	0.96
	No. 12	0.95	0.95
No. 11	No. 10	1.00	0.97
	No. 12	0.99	0.95
No. 12	No. 10	0.99	0.97
	No. 11	0.97	0.97
Mean value		0.98	0.96
